# Eco-Friendly Straws: A Fusion of Soy Protein Isolate and Cassava Starch Coated with Beeswax and Shellac Wax

**DOI:** 10.3390/polym16131887

**Published:** 2024-07-01

**Authors:** Wissuta Choeybundit, Thomas Karbowiak, Aurélie Lagorce, Kittaporn Ngiwngam, Rafael Auras, Pornchai Rachtanapun, Duangjai Noiwan, Wirongrong Tongdeesoontorn

**Affiliations:** 1 School of Agro-Industry, Mae Fah Luang University, Tasud, Chiang Rai 57100, Thailand; 6351407002@lamduan.mfu.ac.th (W.C.); 6671401004@lamduan.mfu.ac.th (K.N.); 2 Research Center of Innovation Food Packaging and Biomaterials Unit, Mae Fah Luang University, Tasud, Chiang Rai 57100, Thailand; 3 Institut Agro Dijon, PAM UMR 02 102, Université Bourgogne Franche-Comté, 1 Esplanade Erasme, 21000 Dijon, France; thomas.karbowiak@agrosupdijon.fr (T.K.); aurelie.lagorce@agrosupdijon.fr (A.L.); 4 School of Packaging, Michigan State University, 448 Wilson Rd, East Lansing, MI 48824, USA; aurasraf@msu.edu; 5 Division of Packaging Technology, Faculty of Agro-Industry, Chiang Mai University, Chiang Mai 50100, Thailand; pornchai.r@cmu.ac.th; 6 The Cluster of Agro Bio-Circular-Green Industry (Agro BCG), Chiang Mai University, Chiang Mai 50100, Thailand; 7 Center of Excellence in Materials Science and Technology, Chiang Mai University, Chiang Mai 50200, Thailand; 8 Department of Postharvest Technology, Faculty of Engineering and Agro-Industry, Maejo University, Chiang Mai 50290, Thailand; duangjai_nw@mju.ac.th

**Keywords:** sustainable straw, edible biopolymer, wax coating, mechanical strength

## Abstract

This research aimed to produce eco-friendly straws using soy protein isolate (SPI) and cassava starch (CS) at different ratios by the extrusion technique and by coating with beeswax and shellac wax. Three straw formulations (F) (F1: 24.39% SPI–24.39% CS; F2: 19.51% SPI–29.37% CS; and F3: 14.63% SPI–34.15% CS) were prepared, incorporating glycerol (14.6% *w/w*) and water (36.6% *w/w*). After extrusion and drying at 80 °C for 20 h, visual assessment favored F2 straws due to smoother surfaces, the absence of particles, and enhanced straightness. For the physical property test, the straws were softened in pH buffer solutions for 5 min. To simulate practical application, mechanical bending strength was studied under different relative humidity (RH) settings. Water absorption reduced the strength as RH increased. F2 straws outperformed other formulations in bending strength at 54% RH. For hydrophobic coatings, F2 was chosen. Beeswax- and shellac wax-coated straws displayed negligible water absorption and sustained their integrity for over 6 h compared to uncoated straws. This study shows that extrusion and natural coatings may make sustainable straws from SPI and CS. These efforts help meet the growing demand for eco-friendly plastic alternatives, opening up new options for single-use straws.

## 1. Introduction

Thailand and other Asian countries, recognizing the urgent need to combat plastic pollution, have initiated measures to curtail single-use plastics [1]. By setting targets to phase out items like plastic bags, Styrofoam containers, and straws from national parks and public spaces, the country aimed to reduce the environmental impact and white pollution of single-use plastic [2]. These steps are part of a global movement where various nations, including the European Union, have taken decisive actions against single-use plastics. Many regions and cities worldwide have either implemented bans or introduced taxes to discourage the use of these items, striving to mitigate the environmental harm caused by plastic waste [3]. Though specific regulations differ across regions, the overarching goal remains consistent to minimize plastic pollution and encourage the adoption of eco-friendly alternatives.

The use of single-plastic packaging has been predominantly influenced by the rise of population growth, urban expansion, and increased consumption [4]. However, there is a growing demand for eco-friendly packaging in the ready-to-eat food industry, food delivery services, and industrial waste management systems [5]. Biodegradable and biobased polymers, such as plant protein including soy protein isolate, starch, cellulose, and polylactic acid (PLA), have gained significant attention as they are biobased, inherently safe for consumers, and cost-effective [6]. While pure PLA or PLA composites are not readily biodegradable and require industrial composting, soy protein has emerged as a promising raw material for developing biobased and biodegradable packaging materials [7].

Biodegradable straws are made from various natural and renewable materials, each offering unique benefits. Polylactic acid (PLA), derived from corn starch or sugarcane, is compostable under industrial conditions [8]. Polyhydroxyalkanoates (PHAs) are produced by bacterial fermentation and are fully biodegradable in multiple environments [9]. Paper straws, often coated for durability, are both biodegradable and compostable [10]. Bamboo provides a strong, reusable option [11], while starch-based plastics from corn, potato, or cassava are blended for enhanced properties [12]. Bagasse, a sugarcane byproduct, offers good mechanical strength [13]. Rice and wheat stalks, agricultural byproducts, are suitable for molding into straws [14]. Chitosan, from crustacean shells, has antimicrobial properties [15], and algae-based materials are a renewable option with lower environmental impact [16]. Soy protein isolate and cassava starch combine biodegradability and flexibility, making them ideal for straws [17].

One promising area of research is the development of biodegradable straws using plant-based materials such as soy protein isolate (SPI) and cassava starch (CS). Both SPI and CS are derived from renewable resources and possess the necessary properties to form biodegradable films and products. Numerous studies have investigated the utilization of soy protein isolate (SPI) and cassava starch (CS) in the formation of biodegradable films. However, there is a notable dearth of research exploring their application in the production of drinking straws, which underscores the primary motivation for our investigation into both materials. SPI, derived from soybeans, exhibits exceptional film-forming properties and mechanical strength, while CS contributes flexibility and biodegradability to composite materials [18]. In 2023, the global market for SPI was estimated to be 4.2 million tons, while the production of CS was estimated to be 6.9 million tons. SPI and CS are extensively utilized in several industries across multiple sectors.

Soy protein possesses favorable properties, such as compatibility with various materials, home-composting ability, large-scale production, edibility, consumer safety, and cost-effectiveness [19]. Researchers have explored different forms of soy protein, including defatted soy flour (DSF), soy protein concentrate (SPC), and soy protein isolate (SPI), which contain varying protein contents [20]. While soy protein can be molded into products like edible films and plant pots, it does face limitations in terms of strength, melt flow index, and water absorption [21]. SPI, valued for its protein content and functional properties, is widely used in meat and dairy alternatives, baked goods, and processed foods. However, blending soy protein with starch-based edible biopolymers can improve these properties in the food industry [22]. CS provides texture and stability in baked goods, snacks, dairy products, and gluten-free foods [23]. The absence of research in straw production using SPI and CS blends highlights a critical gap in the current literature. SPI and CS play crucial roles in the food industry.

Extrusion is an efficient process widely employed in industrial applications for packaging material and food production [24]. The proper selection of processing parameters is crucial for extrusion, as they significantly influence the properties of biopolymer packaging materials [25]. One of the limitations of biopolymers for packaging materials is their susceptibility to water, which affects their moisture resistance. Consequently, enhancing the moisture resistance of biopolymer materials has been a focal point in numerous studies and research efforts [26]. Among the environment-friendly substances used for surface coating in packaging applications, proteins, polysaccharides, lipids, and waxes are the most common biomaterials [27]. Hydrophobic organic compounds with medium-length chains, such as beeswax and shellac wax derived from various sources like plants, insects, and marine life, find numerous applications as biobased coating materials [28]. 

This study focuses on using extrusion to develop biocomposites based on soy protein isolate, expanding their potential applications in the market. Specifically, biodegradable straws were produced by blending SPI and cassava starch (CS), a novel approach that, to the best of our knowledge, has not been previously explored. The production of soy protein-based biodegradable straws offers several benefits, such as reducing plastic waste and adding value to agricultural raw materials, thereby bringing environmental and economic advantages to developing more sustainable food-grade straws. The main objective of this work was to investigate the best conditions for producing SPI-CS blended straws using the extrusion technique followed by coating with beeswax and shellac wax for improved mechanical and water resistance properties. By achieving this, the study aims to contribute to developing eco-friendly and economically viable food-grade straws while reducing the environmental impact of plastic waste.

## 2. Material and Methods

### 2.1. Procurement of Raw Materials

SPI was purchased from Bulk Co., Ltd. in London, England, and glycerol (food grade) from Cooper Co., Ltd., Melun, France, was used as a plasticizer. Chemical analysis indicated that the fat, carbohydrates, fiber, salt, and protein content of SPI were 0.4%, 3.5%, 0.2%, 2.6%, and 85%, respectively; all percentages are on a wet basis. Thai World Import & Export Co., Ltd. in Bangkok, Thailand, supplied cassava starch. Beeswax from Centifolia in Nueil-les-Aubiers, France, was used for the coating preparation, and Excelacs Co., Ltd. in Bangkok, Thailand, gifted shellac.

### 2.2. Formulation and Production of SPI-CS Straws

**Figure 1:**
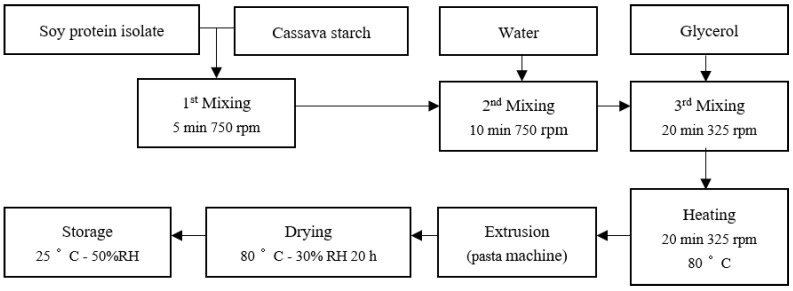
Flow diagram of SPI-CS straw production.

The straw formulation consisted of four ingredients: SPI, CS, glycerol, and water. Three formulations (Table 1) were prepared with varying SPI and CS content. The water and glycerol content were the same for all formulations, at 36.6% and 14.6%, respectively. Making the SPI-CS straw involved several unit operations, as shown in Figure 1. The dry ingredients (SPI and CS) were initially mixed using a cutter machine (model: Stephan UMM/SK, Germany) at 750 rpm for 5 min. Then, water was added, and a second mixing step was performed at 750 rpm for 10 min. Subsequently, glycerol was added, and a final mixing was conducted at 325 rpm for 20 min, ensuring thorough and complete mixing upon a virtual check with a human eye. The resulting dough was heated while mixing at 325 rpm for 20 min at 80 °C. Finally, the hot dough was extruded using a pasta machine (Rotisol, Chelles, France) equipped with a hollow cylinder die of 0.5 cm diameter. The pasta straw samples were then dried in a climate-controlled chamber (model: VOTSCH VC4060 Votsch, Balingen, Germany) at 80 °C for 20 h, maintaining a relative humidity of 30%.

### 2.3. Coating of SPI-CS Straws with Beeswax and Shellac Wax

A total of 48.05 g of beeswax was weighed in a glass beaker and melted in a water bath at 70 °C to achieve a final volume of 50 mL. The SPI-CS straws, measuring 3 cm in length, were then coated by immersing them in the melted beeswax solution. The shellac coating solution was prepared by dissolving 15% (*w/v*) shellac in 95% ethanol, and 2% (*w/v*) oleic acid was added as an emulsifier, following the method described by Farag and Leopold (2011) [29]. The SPI-CS straws were cut to a length of 3 cm, and the coating was carried out by immersing them in different coating solutions. SPI-CS straw samples coated with beeswax and shellac were allowed to dry at room temperature overnight (~25 °C; relative humidity ~50%).

### 2.4. Characterization of SPI-CS Straws without and with a Coating of Beeswax and Shellac

#### 2.4.1. Moisture Sorption Isotherm

The moisture sorption isotherms (MSIs) of the SPI-CS coated and uncoated straw samples were determined at 25 °C using the method described by Labuza (1975) [30]. Initially, the straws underwent conditioning at 25 °C and 50% relative humidity for 24 h. Following conditioning, each straw sample was placed in a separate desiccator containing a saturated salt solution. These solutions included Lithium Chloride (LiCl), Potassium Acetate (KC_2_H_3_O_21_.5H_2_O), Magnesium Chloride (MgCl_2_), Potassium Carbonate (K_2_CO_3_), Magnesium Nitrate (Mg(NO_3_)_2_), Potassium Iodide (KI), Sodium Nitrate (NaNO_3_), Potassium Chloride (KCl), and Potassium Nitrate (KNO_3_) to set relative humidity values of 11%, 23%, 33%, 44%, 53%, 68%, 74%, 84%, and 94%, respectively. These salt solutions established specific equilibrium water activities (*a*_w_) ranging from 0.11 to 0.92, based on Greenspan’s method (1977). After the equilibration process, the straw samples were periodically weighed until reaching an equilibrium moisture content for each specific *a*_w._

#### 2.4.2. Water Absorption Measurement

The evaluation of the water absorption of the SPI-CS coated and uncoated straw samples stored at 25 °C and 50% RH for 24 h and placed in a desiccator was conducted by immersing 3 cm lengths of each sample in 30 mL of buffer solution, Mcllvaine’s citric-phosphate buffer, at pH 2, pH 5, and 8 for 5, 10, 15, and 20 min. Before immersion, the samples were weighed to determine their initial weight (*Wi*). After the specified immersion times, the excess buffer solution was removed using compressed air, and the final weight of the samples was measured (*Wt*). The water absorption percentage was calculated using the method outlined by ASTM D570 as shown in Equation (1):

Water absorption (%) = ((*Wt* − *Wi*)/*Wi*) × 100 (1)

where *Wi* = initial weight of the sample (g); *Wt* = final weight after immersion (g).

#### 2.4.3. Mechanical Properties

The fracture properties of both the SPI-CS coated and uncoated straw samples were assessed using the three-point bending test. The testing was conducted at different equilibrium water activities (*a*_w_) and a constant temperature of 25 °C. The evaluation was performed using a texture analyzer TA-HD+ from Stable Micro Systems in Godalming, Surrey, UK. The testing method followed the procedure outlined by [31]. SPI-CS straw samples of 5 cm in length were positioned on the machine, with the lower supporting blades set at 3 cm apart. The straws were subjected to fracture by the blade, which moved at 3 mm/s. To measure the force exerted during the test, a 30 kg load cell was utilized. Each sample was replicated ten times to ensure the accuracy and reliability of the results.

#### 2.4.4. Microstructure

Micrographs of both the SPI-CS coated and uncoated straw samples were obtained using a scanning electron microscope (SEM) model JEOL JSM-6700F from Tokyo, Japan. The samples were stored at 25 °C and a relative humidity of 50% before observation. A thin layer of gold was sputtered onto the samples to enhance the imaging quality and prevent sample charging during the SEM analysis, following the method outlined by [32].

### 2.5. Statistical Analysis

The data were subjected to analysis of variance (ANOVA) using IBM SPSS Statistics 20 to determine if there were statistically significant (*p* ≤ 0.05) differences in each attribute. The Duncan new multiple range test was used to determine which samples differed significantly.

## 3. Results and Discussion

### 3.1. Physical Characteristics SPI-CS Straws from Different Formulations

**Figure 2:**
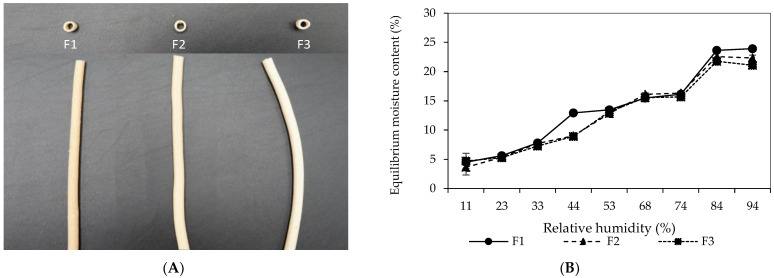
Photographs of SPI-CS straw produced from different formulations (**A**) and moisture sorption isotherm of SPI-CS straws prepared from different formulations (**B**). Key caption about formulations from Table 1.

The impact of different formulations (F1, F2, and F3), summarized in Table 1, on the physical properties of SPI-CS straws was investigated. Formulation F1, with equal SPI and CS percentages (24.4%), exhibited a rough surface structure. This aligns with the existing literature, suggesting that higher SPI content can lead to surface-visible protein particles due to ionic bonding lowering the net charge of the particles [33]. Formulation F2, with increased CS content (29.3%), displayed smoother surfaces compared to F1, indicating the reduced presence of SPI particles and improved smoothness due to higher CS content, as shown in Figure 2A. Formulation F3, containing the highest CS content (34.2%), also showcased a smooth surface; however, the straws appeared curved, unlike F1 and F2. This curvature might be attributed to the higher CS content causing structural changes, potentially displacing SPI and creating holes in the protein network. Incorporating SPI into CS can form a protein–starch composite [34]. At lower SPI levels, as in F2, the protein dispersion in the composite displayed a uniform structure. However, increasing the SPI content (F1) reduced this dispersion, in line with earlier research indicating the influence of SPI concentration on dispersion [34]. The reduction in SPI particles with increased CS content (F2) and the smooth surfaces in F3 (higher CS) was observed to blend well. Conversely, SPI on F1 surface and the curvature of F3 showed segregated particles. This further implies that SPI-CS combinations could form gel-like structures. Changes in surface appearance and curvature might be due to alterations in the internal gel structure due to varying compositions of SPI and CS.

### 3.2. Effects of Water on the Mechanical Properties of SPI-CS Straws

Figure 2B illustrates the relationship between equilibrium moisture content and water activity (*a*_w_) of uncoated straws prepared from different formulations. This relationship demonstrated a J-shaped isotherm, which showed characteristics of a type III isotherm similar to those observed elsewhere for cassava starch–soy protein concentrate films [35]. Initially, as *a*_w_ increased, the moisture sorption rate of the straws also increased. Notably, the straws exhibited relatively low moisture sorption at low *a*_w_ levels. However, a significant rise in moisture content occurred when the relative humidity exceeded 74%. This led to marked changes in the physical properties of the straws, rendering them unsuitable for usage due to altered characteristics. The incorporation of plasticizers enhanced straw hydrophilicity by exposing hydroxyl groups. Similar findings were reported by [36], who observed increased moisture content in whey protein films incorporated with tarbush polyphenols and candelilla wax with higher concentrations of glycerol in the formulation. Remarkably, in this study, no significant differences were observed in moisture absorption among the three formulations because of the constant level of water and glycerol concentration.

**Figure 3:**
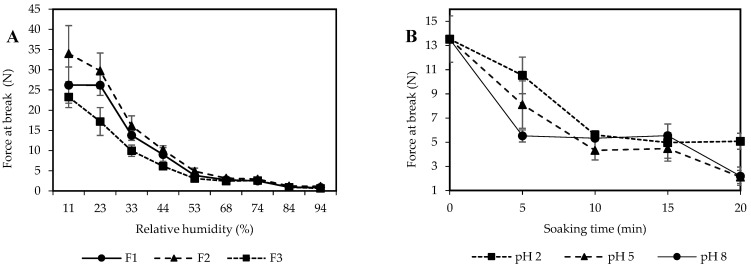
Mechanical property of F1, F2, and F3 straws (**A**) and beeswax-coated F2 straws immersed in pH 2, 5, and 8 solutions with 0–20 min of soaking time at 25 °C (**B**).

The mechanical strength of SPI-CS straws was assessed through the “three-point bending test”, measuring the force required to break the straws. Figure 3A depicts the influence of water activity on the force at break values of uncoated SPI-CS straws prepared from different formulations. An evident decrease in force at break was observed with increasing *a*_w_. At an *a*_w_ of 0.11, the interaction between proteins and starch can occur, which could be evidenced by Fourier transform infrared spectroscopy (FTIR), X-ray diffraction (XRD), and dynamic mechanical analysis (DMA) [37].

### 3.3. Effect of a Beeswax or Shellac Wax Coating in the Physical and Mechanical Strength of SPI-CS Straws

**Figure 4:**
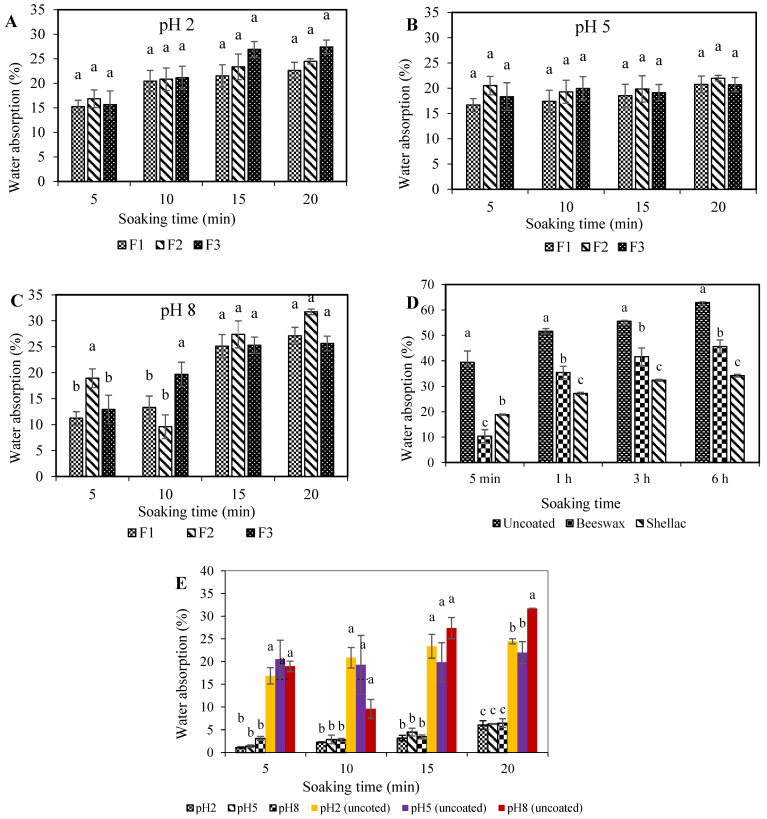
Water absorption of uncoated SPI-CS straws at pH 2 (**A**), pH 5 (**B**), pH 8 (**C**), beeswax- and shellac-coated F2 straws (**D**), and F2 uncoated and coated straws (**E**) immersed in aqueous solutions of pH 2, 5, and 8 at 25 °C.

The water absorption behavior of uncoated SPI-CS straws across different pH solutions (pH 2 and pH 5) and time intervals (5, 10, 15, and 20 min) is depicted in Figure 4A–C. Notably, no significant (*p* > 0.05) difference was observed in water absorption between straws immersed in pH 2 and pH 5 solutions. At pH 2, the water absorption ranged from 15.3% to 27.4% over 5 to 20 min. Similarly, pH 5 buffer solutions displayed water absorption percentages ranging from 16.7% to 22.0% (Figure 4A). Water absorption values remained relatively consistent from 5 to 20 min for pH 5 (Figure 4B). Remarkably, the highest water absorption was recorded at pH 8, peaking at 31.7% after 20 min (Figure 4C). Over 5 and 10 min, significant (*p* ≤ 0.05) differences were observed among the three formulations. Formulation 2 (F2) exhibited higher water absorption than F1 and F3 at 5 min, while F3 displayed the highest water absorption at 10 min. Extreme pH conditions cause electrostatic repulsions between proteins, affecting their interactions Zhou [33]. The wet shear strength of SPI was optimal around its isoelectric point (pH = 4.5), with conditions close to the isoelectric point (pI) inducing structural alterations and protein aggregation [38].

A comparison of water absorption among uncoated, beeswax-coated, and shellac-coated straws is illustrated in Figure 4D. Uncoated straws exhibited higher water absorption than their coated counterparts. Notably, shellac-coated straws demonstrated lower water absorption than beeswax-coated ones after 5 min. This outcome aligns with the findings of [39], highlighting shellac’s superior hydrophobicity. Water absorption analysis for F2 straws coated with beeswax at pH 2, 5, and 8 is displayed in Figure 4E. These coated straws exhibited water absorption between 1% and 7% (*w/w* wet basis) after 5 min of soaking, gradually reaching a maximum of around 7% after 20 min. In comparison, uncoated straws absorbed over 27% water after 20 min. The water resistance of beeswax can be attributed to its composition of long-chain alcohols, fatty acids, and their esters [40].

Visual observations indicated that F2 straws softened after 5 min of soaking, rendering them unsuitable for drinking. The application of a coating could mitigate or delay water sorption. The texture measurement (three-point bending test) remained feasible after 20 min of soaking (Figure 3B), indicating that the straws maintained rigidity even with coatings. Before soaking, the force at break was approximately 14 N. As soaking progressed, straws softened, with forces dropping to 2 N at pH 5 and 8 and 5 N at pH 2. Despite softening, the straws retained measurable rigidity.

### 3.4. Microstructure of Uncoated and Coated SPI-CS Straws

**Figure 5:**
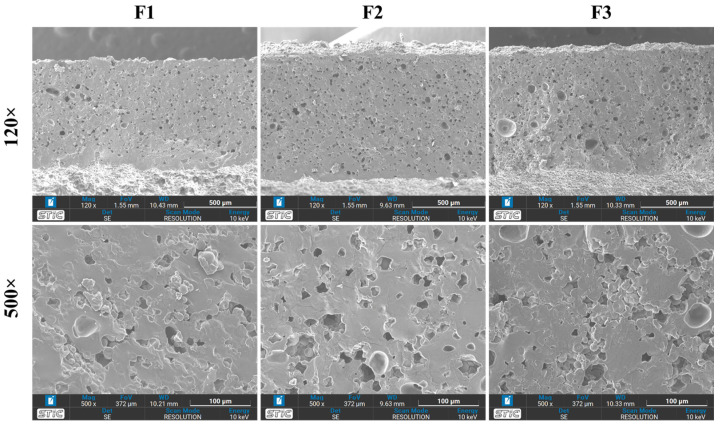
Micrographs of SPI-CS uncoated straws of different formulations at magnifications of 120× and 500×.

Figure 5 presents micrographs illustrating the microscopic structure of uncoated SPI-CS straws at varying magnifications (120× and 500×). Upon observation, the surface cross-section of these SPI-CS straws displayed a smooth structure across different points. Although tiny holes were present, the surface displayed notable smoothness without roughness or unevenness. Notably, an increase in SPI content reduced both the number and size of these holes. This outcome suggests that the heightened SPI concentration contributed to a higher cross-linking density within the material, leading to decreased non-uniformity and a smoother surface. This enhanced surface smoothness is crucial for reinforcing the water resistance capability of the material. Remarkably, this finding is in harmony with prior research [41], which revealed that microstructures of novel composite films based on SPI and oilseed floor were smoother compared to control films [42].

**Figure 6:**
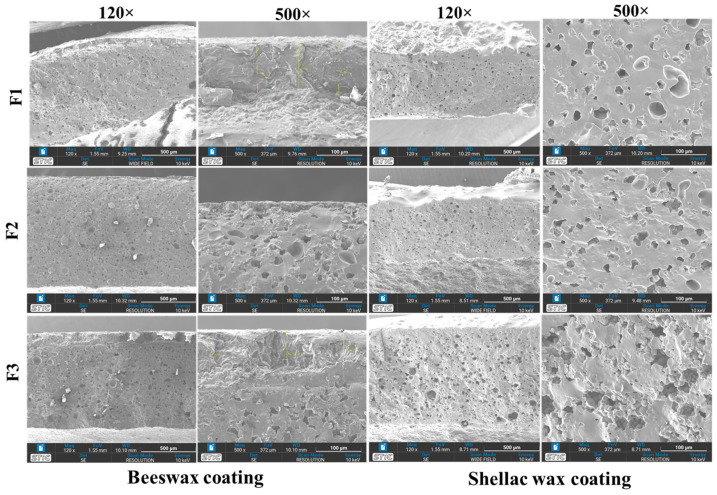
Micrographs of SPI-CS beeswax- and shellac-coated straws of different formulations at magnifications of 120× and 500×.

Micrographs of SPI-CS straws coated with beeswax and shellac wax are displayed in Figure 6, showing their microscopic structure at various magnifications (120× and 500×). Post-coating, the sample surface exhibited evident smoothness. Beeswax film coating effectively covers the straw’s surface and bridges gaps between SPI and CS components, significantly enhancing water resistance. Zhang and Huining [43] reported on beeswax latex modified with a guanidine-based antibacterial polymer coating on a paper surface, augmenting water resistance. The shellac wax coating yielded a smooth, intact structure, exhibiting robust adhesion and continuity with microfibrillar cellulose, enhancing its barrier strength significantly [44]. These micrographs collectively highlight the transformative impact of beeswax and shellac wax coatings on the surface properties of SPI-CS straws. These coatings contribute to enhanced water resistance and structural integrity, offering valuable insights into material modifications that are pivotal for developing eco-friendly alternatives to conventional plastic straws. Subsequent research will further develop the water resistance of SPI-CS straws.

## 4. Conclusions

In this study, biodegradable straws were developed using a combination of SPI and CS in different formulations. The preparation involved mixing the components using a cutter machine, followed by extrusion through a pasta machine. The influence of varying SPI content on the straw’s surface was evident, as excessive protein content hindered complete dispersion when combined with starch. The best results were obtained when SPI and CS were present in a 40:60% *w/w* (F2) ratio, achieving a balanced composition. Interestingly, the different concentrations of SPI and CS did not significantly impact water absorption across various pH buffer solutions. Notably, at pH 5, SPI-CS straws exhibited lower water absorption than other pH levels. The assessment of breaking force revealed that F2, containing a midpoint level of SPI and CS, demonstrated superior strength compared to other formulations at the same water activity level. Following 5 min of soaking, the straws experienced softening, rendering them unsuitable for use. However, the application of beeswax and shellac wax coatings showcased the potential to enhance the water resistance of the straws. These findings indicate that when coated with natural waxes, compostable SPI-CS straws hold promise as eco-friendly alternatives for single-use straws in beverage applications. Further research will be conducted to further develop the water resistance of SPI-CS straws.

## Figures and Tables

**Table 1:** Composition of SPI-CS straw formulations.

Samples	SPI (%)	CS (%)	Glycerol (%)	Water (%)
F1	24.4	24.4	14.6	36.6
F2	19.5	29.3	14.6	36.6
F3	14.6	34.2	14.6	36.6

SPI: soy protein isolate; CS: cassava starch; and F1, F2, and F3 denote different formulations.

## Data Availability

All the data are available within the article.
